# Supplementary material for: Perforin-2 clockwise hand-over-hand pre-pore to pore transition mechanism
Source: Nat Commun. 2022 Aug 26;13:5039. doi: 10.1038/s41467-022-32757-4 (PMC9418332; doi:10.1038/s41467-022-32757-4)
Supplement: Supplementary file 1 — Supplementary Information [file 41467_2022_32757_MOESM1_ESM.pdf]

Title

Perforin-2 clockwise hand-over-hand pre-pore to pore transition mechanism

Author List

Fang Jiao,[1,2,3,*] François Dehez,[4] Tao Ni,[5] Xiulian Yu,[5,6] Jeremy Dittman,[7] Robert Gilbert,[5,6] Christophe Chipot,[4,8] Simon Scheuring[1,2,9,*]

Affiliations

[1] Department of Anesthesiology, Weill Cornell Medicine, New York City, NY 10065, USA
[2] Department of Physiology and Biophysics, Weill Cornell Medicine, New York City, NY 10065, USA
[3] Laboratory of Soft Matter Physics, Institute of Physics, Chinese Academy of Sciences, Beijing 100190, China
[4] Laboratoire International Associé, Centre National de la Recherche Scientifique et University of Illinois at Urbana-Champaign, Unité Mixte de Recherche no 7019, Université de Lorraine, Vandœuvre-lès-Nancy cedex, France
[5] Division of Structural Biology, Wellcome Centre for Human Genetics, University of Oxford, Roosevelt Drive, Oxford OX3 7BN, UK
[6] Calleva Research Centre for Evolution and Human Sciences, Magdalen College, University of Oxford, Oxford OX1 4AU, UK
[7] Department of Biochemistry, Weill Cornell Medicine, New York City, NY 10065, USA
[8] Department of Physics, University of Illinois at Urbana-Champaign, Urbana, IL 61801, USA
[9] Kavli Institute at Cornell for Nanoscale Science, Cornell University, Ithaca, New York 14853, USA

* Correspondence to: Fang Jiao (fang.jiao@iphy.ac.cn) and Simon Scheuring (sis2019@med.cornell.edu)

Supplementary Information

Supplementary Figure 1:

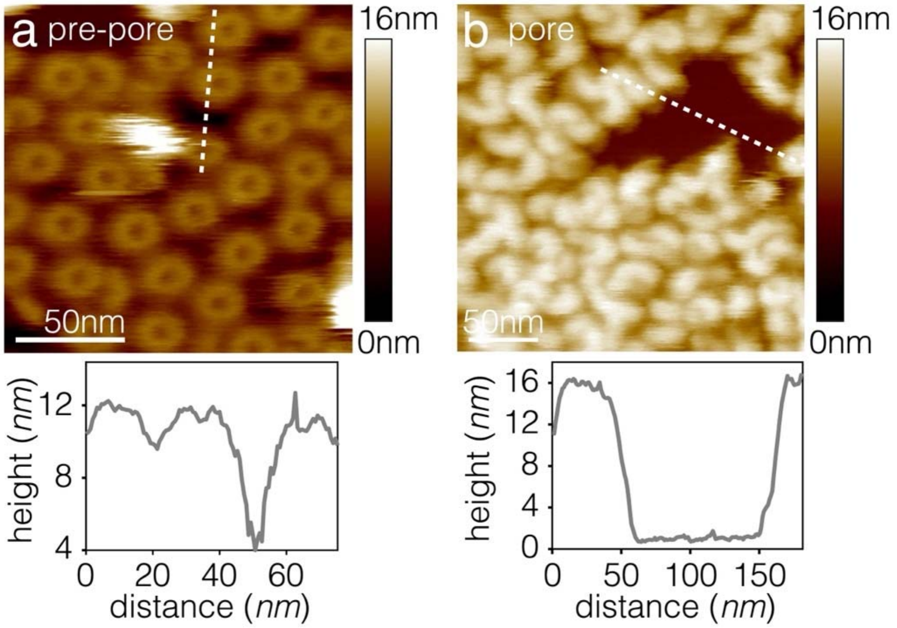

Supplementary Figure 1 | HS-AFM images of PFN2 pre-pore and pore oligomers on and in a *E. coli* lipid bilayer membrane. (**a**) PFN2 pre-pore oligomers protrude by ~8.2 nm on top of an *E. coli* lipid membrane as reported by the section analysis (bottom, see also figure 1l in the main text), reaching a total height of ~12 nm above mica. In this experiment, only half of the typical PFN2 concentration, ~0.021 mg/ml, was used and the incubation time was shortened to 2.5 minutes for a lower packing density of the pre-pore rings giving access to the membrane level in between the rings (experimental repeats, *n* = 3). (**b**) PFN2 pore oligomers have a total height of ~16 nm as reported by the section analysis (bottom). The imaging region was selected for an area with a defect in the *E.* coli lipid bilayer to measure the full height of the pore assemblies (see figure 1f) (experimental repeats, *n* = 3).

Supplementary Figure 2:

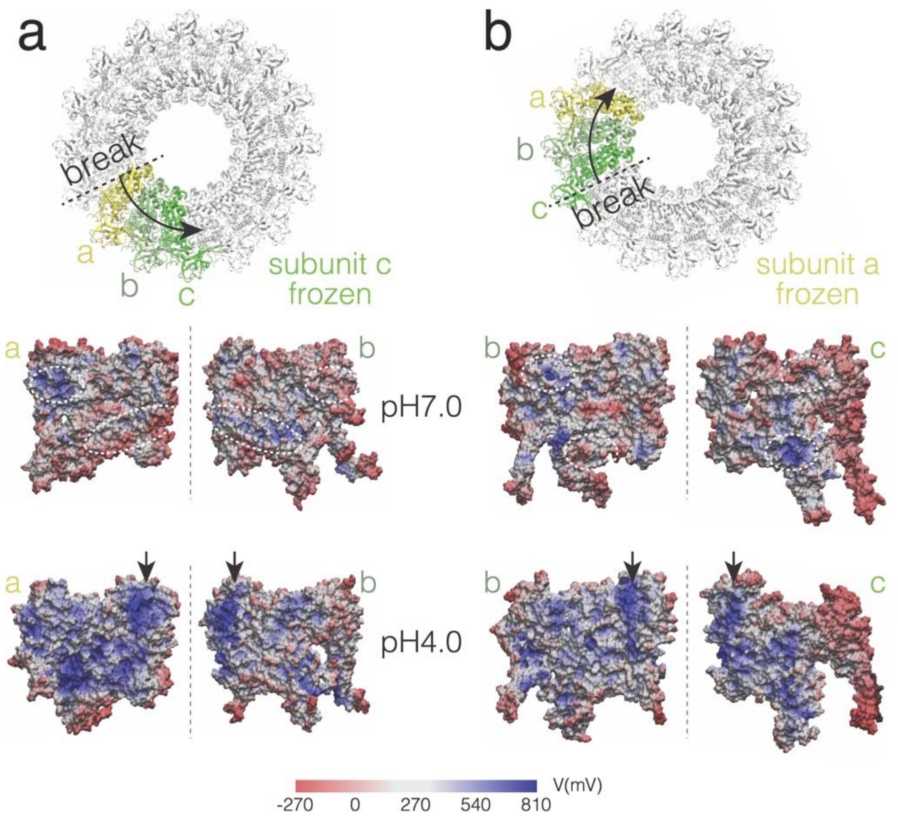

Supplementary Figure 2 | The PFN2 pre-pore subunit interface between adjacent subunits. Top view of the PFN2 pre-pore structure (PDB 6SB3) with subunit-a, -b and -c illustrated and labeled, as well as the location of the break in the pre-pore ring defining the position of the free subunit and the consequential CCW (**a**) and CW (**b**) directionality of the analysis (top). Electrostatic surface representations of the interfaces of subunits a and b (**a**) and b and c (**b**) at neutral pH (middle) and acidic pH (bottom). Charge complementarities between the subunits at neutral pH are highlighted by the dashed outlines. Due to protonation of titratable residues, these charge complementarities are lost at acidic pH. Note the conformational rearrangement of the CCT domain in subunit a between pH7 and pH4 (see Supplementary Figure 4).

Supplementary Figure 3:

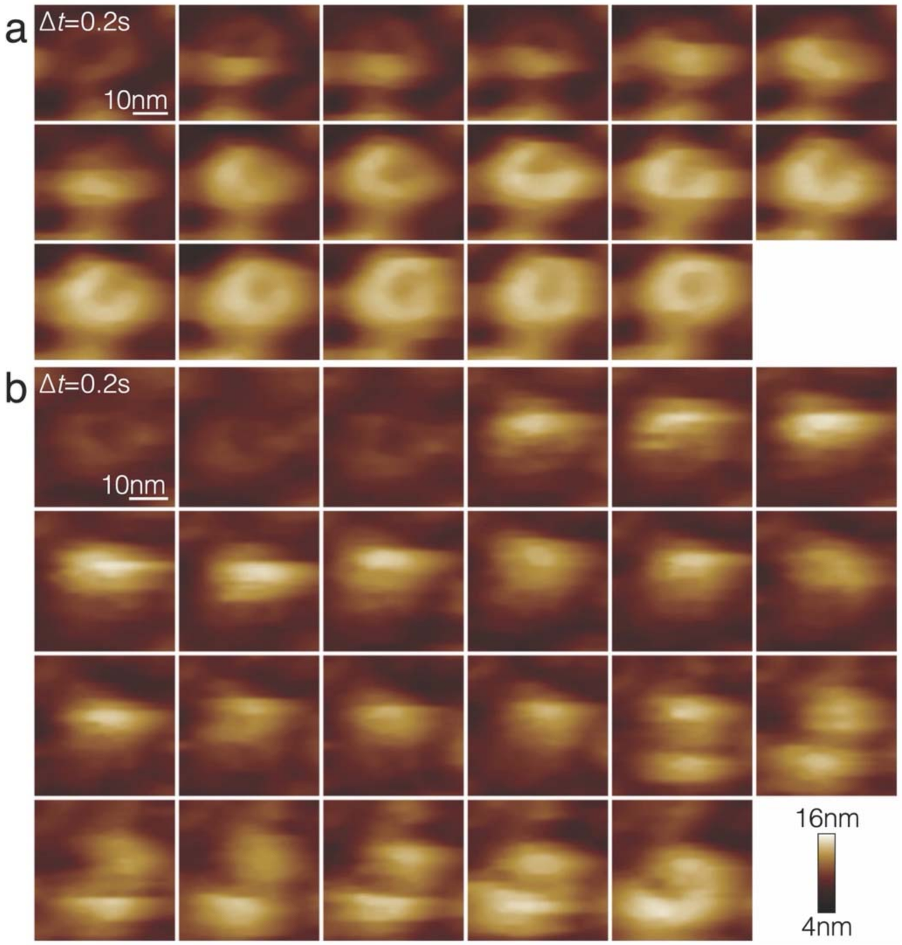

Supplementary Figure 3 | HS-AFM imaging of particular PFN2 pre-pore to pore transition examples. (**a**) PFN2 full ring pre-pore to full ring pore transition. (**b**) PFN2 pre-pore-to-pore transition where the pre-pore displayed two breakages and transited into two small pore arcs separately that later fused into one larger pore arc.

Supplementary Figure 4:

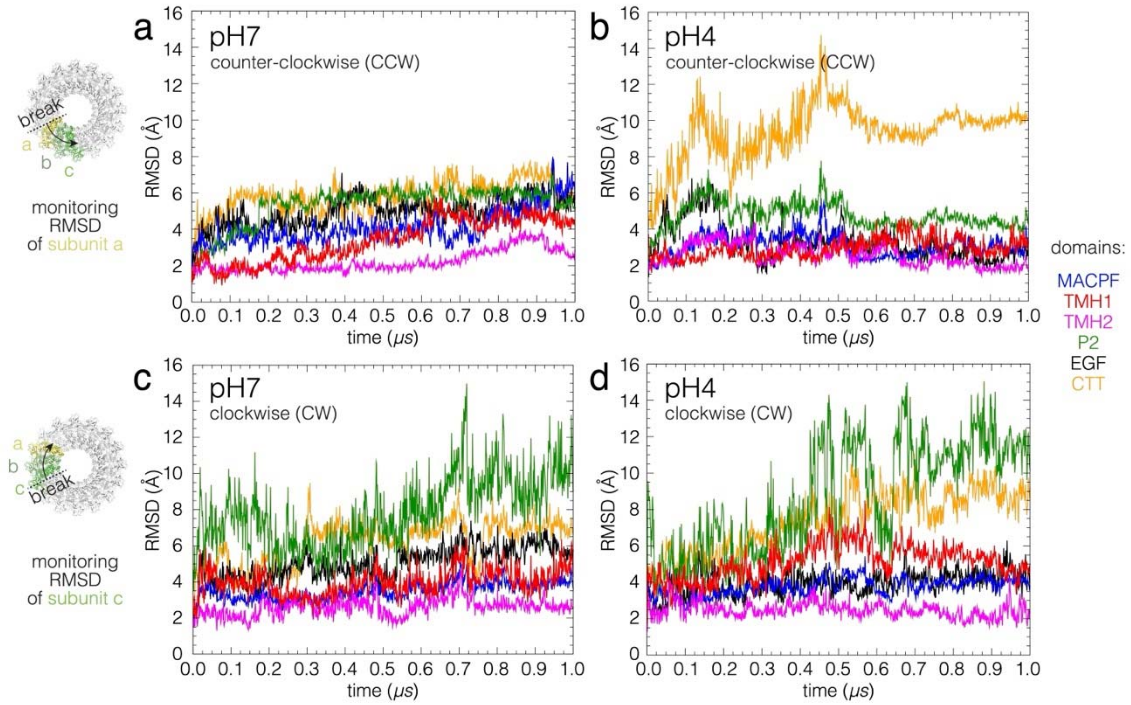

Supplementary Figure 4 | Structural fluctuations of the free subunits upon pre-pore ring breakage by domain. Comparisons of the backbone atom distance root-mean-square deviations (RMSDs) of the various PFN2 domains in the CCW (subunit-a, **a,b**) and the CW (subunit-c, **c,d**) subunits with respect to their initial structures at neutral (**a,c**) and acidic (**b,d**) pH. The CTT domain that extends from the subunit core is also flexible in the CCW setting at pH4 (b, see Supplementary Figure 2). In the CW setting the P2 and CTT domains also display increased fluctuations at pH7 (c), which is observed in the used open ended PFN2 trimer subunit setting; however, in experiment, prepore ring breakages a t pH7 we not detected. Source data are available as a Source Data file.

Supplementary Figure 5:

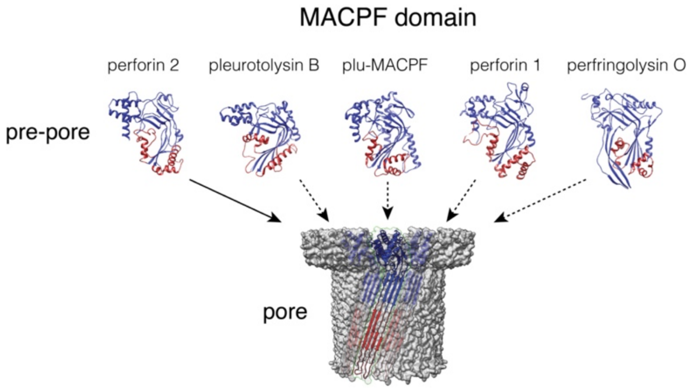

Supplementary Figure 5 | Structural conservation of MACPF proteins. Top: MACPF domain structures of representative MACPF/CDC superfamily proteins: murine perforin 2 (PDB 6SB3), pleurotolysin B (PDB 4OEJ), Plu-MACF of *P. luminescens* (PDB 2QP2), murine perforin 1 (PDB 3NSJ), bacterial perfringolysin O (PFO, PDB 1PFO). PFN2 pore structure highlighting the MACPF domain packing.

Supplementary Table 1:

pH 7.0		CW (subunit a frozen)				CCW (subunit c frozen)			
		a-b interface		c-b interface		a-b interface		b-c interface	
Salt bridge		direct	bridged	direct	bridged	direct	bridged	direct	bridged
K520	D370	0	0	0	0	33	11	10	13
D448	K329	100	0	84	14	83	14	92	8
D482	R339	5	41	81	14	30	48	63	17
K142	E102	43	26	66	15	64	19	64	15
K251	E290	37	21	54	27	24	5	0	1
E44	K90	63	23	96	3	41	55	87	12
K454	E613	19	19	71	7	40	15	56	21
K435	E634	0	0	35	2	24	7	0	0
R61	E74	0	0	50	37	65	35	39	30
R135	E95	0	0	34	27	26	14	23	35
R274	E95	46	54	46	37	62	24	25	26
R274	E99	1	34	0	3	0	7	0	10
R135	E99	0	0	50	10	14	20	6	23
R589	E118	100	0	0	0	0	0	0	0
E425	K332	14	45	0	0	0	2	1	8
K35	E74	63	10	0	0	0	0	2	1
K595	E335	8	10	0	0	0	0	1	0
Total [a]		7	7	11	4	11	4	8	5
Mean occupancy [b]		65	35	61	32	45	41	56	27

pH 4.0		CW (subunit a frozen)				CCW (subunit c frozen)			
		a-b interface		c-b interface		a-b interface		c-b interface	
Salt bridge		direct	bridged	direct	bridged	direct	bridged	direct	bridged
K520	D370	0	0	0	0	4	32	0	0
D448	K329	0	0	0	0	1	17	1	7
D482	R339	0	7	1	24	0	0	0	2
K142	E102	0	0	6	19	0	0	1	2
K251	E290	6	20	1	4	3	16	0	1
E44	K90	0	0	9	30	2	15	2	35
K454	E613	2	52	0	2	0	0	2	12
K435	E634	0	0	0	0	0	0	0	0
R61	E74	0	0	8	17	0	9	1	37
R135	E95	0	7	0	0	0	0	0	0
R274	E95	2	69	0	0	2	18	0	0
R274	E99	0	22	0	0	0	14	0	0
R135	E99	2	35	0	9	0	9	1	34
R589	E118	0	0	0	0	0	12	0	0
E425	K332	1	18	0	0	0	0	1	10
K35	E74	0	0	0	0	0	0	0	0
K595	E335	7	40	1	13	0	0	0	0
Total [a]		0	6	0	2	0	1	0	3
Mean occupancy [b]		0	40	0	27	0	32	0	35

Supplementary Table 1 | Probability of occurrence (in percent, %) of interfacial salt bridges between subunits at neutral (above) and acidic (below) pH, inferred from µs-timescale molecular dynamics trajectories, with either subunit-a (CW) or subunit-c (CCW) frozen. Direct and water-mediated salt bridges (see Fig. 4) are identified based on the distance criteria of $d \leq 4$ Å and $4 \leq d \leq 6$ Å, respectively, where d stands for the distance separating the carboxylate oxygen atoms of aspartate (D) and glutamate (E) from the nitrogen atoms of lysine (K) and arginine (R). Total number (a) and mean occupancy (b) of salt bridges, either direct or water-mediated, having a probability of occurrence greater than 20%. For the presented analysis only the c-b subunit interface when subunit-a is frozen (CW) and only the a-b subunit interface when subunit-c is frozen (CW) are relevant (highlighted in green).

Supplementary Table 2:

Pre-pore-I height	8.2 ± 0.4 nm (on mica)<br>8.1 ± 0.2 nm (above membrane)
Pre-pore-I inter-subunit opening rate (at neutral pH)	Not observed
Pre-pore-I lifetime (at neutral pH)	>> observation time
Pre-pore-I inter-subunit opening rate (at low pH)	1.8 ± 1.2 s^{-1}
Pre-pore-I inter-subunit closing rate (at low pH)	> 5 s^{-1}
Pre-pore-I lifetime (at low pH)	5 s
Pre-pore-II height	~9.7 nm (above membrane)
Pre-pore-I to pre-pore II Δheight	1.6 ± 0.4 nm
Pre-pore-I to pre-pore II conformational change height increase speed	47.3 ± 5 nm/s
Pre-pore-I to pre-pore II conformational change time	~ 34 ms
Pre-pore-I to pre-pore-II transition rate	~13 s^{-1}
Pre-pore-II lifetime	~75 ms
Pore height	14.0 ± 0.5 nm (on mica)<br>12.1 ± 0.6 nm (above membrane)<br>~16 nm (above mica in membrane)
Pre-pore-II to pore Δheight	2.2 ± 0.2 nm
Pre-pore II to pore conformational change height increase speed	48 ± 7 nm/s
Pre-pore-II to pore conformational change time	~ 46 ms
Pre-pore-II to pore transition rate	~15 s^{-1}
Pre-pore-I to pre-pore-II to pore total transition time	1.3 ± 0.6 s
Pore lifetime	>> observation time

Supplementary Table 2 | Summary of kinetic parameters of the PFN2 pre-pore to pore transition
