# Supplementary material for: Perforin-2 clockwise hand-over-hand pre-pore to pore transition mechanism
Source: Nat Commun. 2022 Aug 26;13:5039. doi: 10.1038/s41467-022-32757-4 (PMC9418332; doi:10.1038/s41467-022-32757-4)
Supplement: Supplementary file 3 — Description of Additional Supplementary Files [file 41467_2022_32757_MOESM3_ESM.pdf]

Description of Additional Supplementary Files

File Name: Supplementary Movie 1
Description: HS-AFM movie of PFN2 pre-pore rings on a *E. coli* lipid bilayer at neutral pH 7.5. The movie is recorded at 1 frame per second (1 s temporal resolution)

File Name: Supplementary Movie 2
Description: HS-AFM movie of PFN2 pre-pore rings on a *E. coli* lipid bilayer at acidic pH 4.0. Pre-pore rings transition into pore arcs in a consistent clockwise (CW) hand-overhand mechanism. The movie is recorded at 5 frames per second (200 ms temporal resolution)
